# Cytotoxicity of selected Cameroonian medicinal plants and *Nauclea pobeguinii* towards multi-factorial drug-resistant cancer cells

**DOI:** 10.1186/s12906-015-0841-y

**Published:** 2015-09-04

**Authors:** Victor Kuete, Louis P. Sandjo, Armelle T. Mbaveng, Jackson A. Seukep, Bonaventure T. Ngadjui, Thomas Efferth

**Affiliations:** Department of Pharmaceutical Biology, Institute of Pharmacy and Biochemistry, University of Mainz, Staudinger Weg 5, 55128 Mainz, Germany; Department of Biochemistry, Faculty of Science, University of Dschang, Dschang, Cameroon; Department of Pharmaceutical Sciences, CCSUniversidade Federal de Santa Catarina, Florianópolis, 88040-900 SC Brazil; Department of Organic Chemistry, Faculty of Science, University of Yaoundé 1, Yaoundé, Cameroon

**Keywords:** *Nauclea pobeguinii*, Cameroon, Cytotoxicity, Multidrug resistant, Resveratrol, Rubiaceae

## Abstract

**Background:**

Malignacies are still a major public concern worldwide and despite the intensive search for new chemotherapeutic agents, treatment still remains a challenging issue. This work was designed to assess the cytotoxicity of six selected Cameroonian medicinal plants, including *Nauclea pobeguinii* and its constituents 3-acetoxy-11-oxo-urs-12-ene (1), *p*-coumaric acid (2), citric acid trimethyl ester (3), resveratrol (4), resveratrol *β-*_D_*-*glucopyranoside (5) and strictosamide (6), against 8 drug-sensitive and multidrug-resistant (MDR) cancer cell lines.

**Methods:**

The resazurin reduction assay was used to evaluate the cytotoxicity of the crude extracts and compounds, whilst column chromatography was used to isolate the constituents of *Nauclea pobeguinii*. Structural characterization of isolated compounds was performed using nuclear magnetic resonance (NMR) spectroscopic data.

**Results:**

Preliminary experiments on leukemia CCRF-CEM cells at 40 μg/mL showed that the leaves and bark extracts from *Tragia benthamii*, *Canarium schweinfurthii*, *Myrianthus arboreus*, *Dischistocalyx grandifolius* and *Fagara macrophylla* induced more than 50 % growth of this cell line contrary to the leaves and bark extracts of *N. pobeguinii*. IC_50_ values below or around 30 μg/mL were obtained with leaves and bark extracts of *N. pobeguinii* towards two and five, respectively, of the 8 tested cancer cell lines. The lowest IC_50_ value was obtained with the bark extract of *N. pobeguinii* against HCT116 *(p53*^*−/−*^*)* colon cancer cells (8.70 μg/mL). Compounds 4 and 6 displayed selective activity on leukemia and carcinoma cells, whilst 1–3 were not active. IC_50_ values below 100 μM were recorded with compound 5 on all 9 tested cancer cell lines as well as with 4 against 7 out of 8 and 6 against 2 out of 8 cell lines.

Collateral sensitivity was observed in CEM/ADR5000 leukemia cells, MDA-MB-231-*BCRP* breast adenocarcinoma cells (0.53-fold), HCT116 (*p53*^*+/+*^) cells, human U87MG.Δ*EGFR* glioblastome multiforme cells to the methanolic bark extract of *N. pobeguinii*, as well as in MDA-MB-231-*BCRP* cells and HCT116 (*p53*^*+/+*^) cells and U87MG.Δ*EGFR* cells (0.86-fold) to compound 5.

**Conclusions:**

The results of this study demonstrate the cytotoxicity of six Cameroonian medicinal plants, *Canarium schweinfurthii*, *Dischistocalyx grandifolius*, *Tragia benthamii*, *Fagara macrophylla*, *Myrianthus arboreus* and *Nauclea pobeguinii*. We also demonstrated the antiproliferative potential of *Nauclea pobeguinii* against drug-resistant cancer cell lines. Resveratrol and its glucoside are the major cytotoxic constituents in the bark of *Nauclea pobeguinii*.

**Electronic supplementary material:**

The online version of this article (doi:10.1186/s12906-015-0841-y) contains supplementary material, which is available to authorized users.

## Background

Malignacies are still a major public concern worldwide and despite the intensive search for new chemotherapeutic agents, treatment still remains a challenging issue. The situation is more complicated by the spread of drug resistance in tumors. Continuous efforts are necessary to boost drug discovery to treat multidrug resistant (MDR) cancer cells. Many factors are involved in MDR, including the over-expression of ABC transporters, particularly breast cancer resistance protein (BCRP) and P-glycoprotein (P-gp) [1], as well as the epidermal growth factor receptor (EGFR) [2–4] and mutations in the p53 tumor suppressor gene [5]. MDR cancer cells are resistant to a variety of chemically unrelated drugs [6–9]. In our previous studies, we documented the cytotoxicity of several secondary metabolites from selected Cameroonian plants against MDR cancer cells [10–15]. In our continous search for potentially antineoplastic agents from Cameroonian medicinal plants, the present study was designed at investigating the cytotoxicity of *Canarium schweinfurthii* Engl. (Burseraceae), *Dischistocalyx grandifolius* C.B. Clarke (Acanthaceae) and *Tragia benthamii* Bak. (Euphorbiaceae), *Fagara macrophylla* Engl. (Rutaceae), *Myrianthus arboreus* P. Beauv. (Moraceae) and *Nauclea pobeguinii* (Pobég. ex Pellegr.) Merr. ex E.M.A. (Rubiaceae). The work was extended to the isolation of the active constituents of *Nauclea pobeguinii.* The above plants are used in Africa to treat many different ailments (Table 1). However, it has been recommended that ethnopharmacological usages such as immune and skin disorders, inflammatory, infectious, parasitic and viral diseases should be taken into account when selecting plants used to treat cancer, since these reflect disease states bearing relevance to cancer or cancer-like symptoms [16, 17].

**Table 1:** Investigated plants, their traditional use, chemical constituents and bioactivities

Species (family); Voucher number* and place of plant’s collection	Parts used traditionally (part used in this study and percentage yield)	Traditional uses	Bioactive or potentially bioactive components	Bioactivities
*Canarium schweinfurthii* Engl. (Burseraceae); 19652/HNC; Bangangté	Bark, seeds, fruits, leaves and roots (bark: 7.36 %)	Insecticide, dysentery, gonorrhea, coughs, chest pains, pulmonary affections, stomach complaints, food poisoning, purgative and emetic, roundworm infections and other intestinal parasites, emollient, stimulant, diuretic, skin-affections, eczema, leprosy, ulcers [40]; diabetes mellitus [41]; colic, stomach pains, pains after child birth, gale [42]; fever, constipation, malaria, sexually transmitted infection and rheumatism [43].	From oil: Limonene, phellandrenes [40]; bark: Triterpenes steroids, saponins, lipids and glycosides [41]; seeds: schweinfurthinol, *p*-hydroxybenzaldehyde, coniferaldehyde, *p*-hydroxycinnamaldehyde, ligballinol, amantoflavone [44]; catechol, dihydroxyphenylacetic acid, tyrosol, *p*-hydroxybenzoic acid, dihydroxynezoic acid, vanilic acid, phloretic acid, pinoresinol, secoisolariciresinol [45]; tannins, cardiacglycosides, balsams, phenols and flavonoids (Uzama et al.*,* 2012), canarene [46].	Chemoprevention of cancer and other oxidative damage-induced diseases: fruit mesocarp oil extract (Atawodi, 2010) and seed kernel oil extract [47]; Antimycobacterial activities: leaves [48]; antimicrobial activities of dichloromethane, ethylacetate and ethanol extracts against gastrointestinal pathogenic bacteria [49].
*Dischistocalyx grandifolius* C.B.Clarke (Acanthaceae) 27646/SRF Cam; Mbouda	Whole plant (whole plant: 4.53 %)	Infectious diseases [50]	Not reported	Not reported
*Fagara macrophylla* Engl. (Rutaceae) 6173/SRF Cam; Mbouda	Bark, leaves and seeds (bark: 8.43 %; leaves: 6.81 %)	Hypertension [51], colds and stomach-ache, fever, malaria [50], cancers [52]	Alkaloids: tembetarineoblongine, magnoflorine, arborinine, nitidine, dihydronitidine, xanthoxoline, 1-Hydroxy-3-methoxy-N-methyl-acridone, *N*-(4 hydroxyphenethyl)octacosanamide, *N*-(4-hydroxyphenethyl)hexacosanamide, *N*-(4-hydroxyphenethyl)decanamide, *N*-vanilloyltyramine, and *N*-[*O*-docosanoylvanilloyl]tyramine, flavonoid: hesperidin [53–56]	Antiplasmodial :bark [55]; Antifeedant: xanthoxoline and 1-hydroxy-3-methoxy-N-methyl-acridone, arborinine, tembetarine and magnoflorine against *Spodoptera frugiperda*, *S. littoralis* and *S. frugiperda* [54]; Low cytotoxicity: seeds extract towards leukemia CCRF-CEM and CEM/ADR5000 cells lines and pancreatic cancer MiaPaCa-2 cell line [52]; Antitumor: nitidine chloride and 6-methoxy-5,6-dihydronitidine [57]
*Myrianthus arboreus* P.Beauv. (Moraceae) 55499/HNC; Bangangté	Bark, leaves (bark: 7.68 %; leaves: 10.37 %)	Dysentery, diarrhea, vomiting, analgesic, antipyretic, heart troubles, pregnancy complications, dysmenorrheal, incipient hernia, boils, toothache, bronchitis, sore throat [58]; headaches, swellings and tumours, diabetes [40]; stomach disorders [59].	Aqueous and methanol extracts: alkaloids, flavonoid, tannins Cyanogenic glycosides, Phytic acid, saponin, anthocyanin, glycoside, carotenoid, oxalate [58, 60].	Antibacterial: against *Klebsiella pneumoniae, Proteus vulgaris, Staphylococcus aureus, E. coli* oxalate [58]; antiplasmodial: against *Anopheles gambiae* [61].
*Nauclea pobeguinii* (Pobég. ex Pellegr.) Merr. ex E.M.A. (Rubiaceae) 32597/HNC; Mbouda	Bark, leaves, roots (bark: 6.55 %; leaves: 6.31 %)	Abortive, stomach ache, infectious diseases [62]; jaundice [63]; fever, diarrhoea, worm, malaria [64].	Nauclefine 1 and 2, strictosamide, carboxystrictosidine, methylangustoline, 3-*O*-*β*-_D_-fucosyl-quinovic-acid, 3-keto-quinovic-acid [62]; angustoline [65].	Antiplasmodial: extract and 3-*O*-*β*-fucosylquinovic acid and 3-ketoquinovic acid against *Plasmodium falciparum* [64].
*Tragia benthamii* Bak. (Euphorbiaceae) 23329/SRF Cam; Mbouda	Whole plant (whole plant: 5.18 %)	Sores, swollen armpit, abortifacient, child delivery promoter [66].	Tannins, saponins, flavonoids, alkaloids, flavonoids [66].	Antiplasmodial: against *Plasmodium berghei* [66].

*(HNC): Cameroon National Herbarium; (SRFC): *Société des Réserves Forestières du Cameroun*

## Methods

### General procedure

Vacuum liquid chromatography (VLC), column chromatography (CC) and thin layer chromatography (TLC) were performed on silica gel 60 (particle size 90 % <45 mm), 200–300 mesh silica gel, and silica gel GF254 (Merck), respectively. Melting points (m.p.) were measured by an Electro thermal IA 9000 digital melting point apparatus (Electro thermal) and are uncorrected. The NMR data were recorded with a Bruker DRX-400 MHz (Bruker). LR-EI-MS were recorded with JEOL mass spectrometer instrument (JEOL). The purity of the molecules was determined by HPLC (Shimadzu HPLC system), using a LiChrospher100 RP-18 (250 × 4 mm, 5 μM) column and MeOH-H_2_O (6:4 and 8:2)/0.1 TEA as mobile phase with detection at 273 nm.

### Chemicals

Doxorubicin 98.0 % from Sigma-Aldrich was provided by the University Pharmacy of the Johannes Gutenberg University-Mainz and dissolved in PBS (Invitrogen) at a concentration of 10 mM. Geneticin >98 % (72.18 mM) was obtained from Sigma-Aldrich.

### Plant material

The plant materials used in this study were the bark of *Canarium schweinfurthii* Engl. (Burseraceae), the whole plant of *Dischistocalyx grandifolius* C.B.Clarke (Acanthaceae) and *Tragia benthamii* Bak. (Euphorbiaceae), the bark and leaves of *Fagara macrophylla* Engl. (Rutaceae), *Myrianthus arboreus* P.Beauv. (Moraceae), *Nauclea pobeguinii* (Pobég. ex Pellegr.) Merr. ex E.M.A. (Rubiaceae). The plant materials were collected in March and April 2013 in Bangangté and Mbouda (west region of Cameroon). They were identified at the National Herbarium in Yaoundé, Cameroon and compared with voucher specimens formerly kept under the registration number (Table 1).

### Extraction

Air-dried plant material (3 kg for the bark of *Nauclea pobeguinii* and 1 kg for other samples) was powdered and extracted with methanol (MeOH; 10 L for the bark of *Nauclea pobeguinii* and 3 L for other samples) for two days. The organic solution was concentrated *in vacuo* to yield a paste (crude extract). The yield of each extract was determined (Table 1) and the samples were kept at 4 °C until further use.

### Isolation of compounds from the bark of nauclea pobeguinii

The crude extract (80 g) was further poured onto distilled water and separated with dichloromethane (DCM) (A), ethyl acetate, EA (B), and n-butanol, n-BuOH (C) under the non-miscible liquid-liquid process. The concentration *in vacuo* of each organic portion afforded fractions A (42 g), B (12 g), and C (28 g), respectively. A column (5 × 60 cm) was used for the purification of fraction A. Silica gel (160 g) column chromatography was prepared and A was eluted under gradient conditions with pure (100 %) hexane (hex) and EA affording 75 fractions of 100 mL each. A colorless powder (1, 10 mg) was obtained from sub-fractions 15–20, while a brown oil (2, 3.5 mg) was isolated from sub-fractions 25–27. A colorless sticky gum (3, 11.2 mg) was further obtained from sub-fractions 50–63. Moreover, fraction B was loaded onto a silica gel (50 g) column (2 cm × 50 cm) and the column was eluted with DCM/EA (98:2, v/v) to give exclusively 2.1 mg of a brownish solid (4). Fraction C was also loaded onto the same column as A using 150 g of silica gel. The column was eluted with pure DCM/MeOH under gradient condition to afford 90 fractions. Sub-fractions 2–10 afforded 5 mg of compound 4, while sub-fractions 30–40 pooled together based on TLC profile gave a colorless solid (5, 5 mg). Similarly, a yellow solid (6, 20 mg) was obtained after filtration of sub-fractions 60–75. These sub-fractions were pooled together based on the TLC profile, after complete evaporation, the solid residue was recrystallized with acetone to give again 6 (15 mg).

### Structural characterization of isolated compounds

**Fig. 1:**
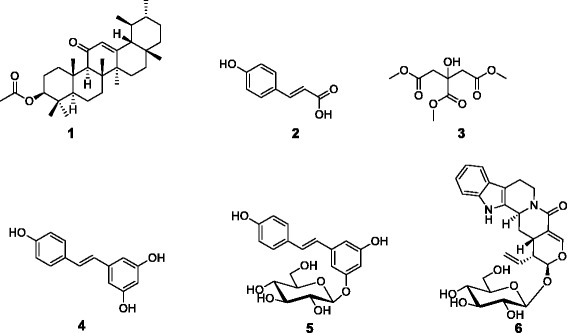
Chemical structures of the compounds isolated from *Nauclea pobeguinii.* **1:** 3-acetoxy-11-oxo-urs-12-ene; **2:** *p*-coumaric acid; **3:** citric acid trimethyl ester; **4:** resveratrol; **5:** resveratrol *β-*_D_ *-*glucopyranoside; **6:** strictosamide

The structures of compounds (1–6) (Fig. 1) were established based on 1D (^1^H, and ^13^C) and 2D (HSQC, COSY and HMBC) NMR spectroscopy as well as mass spectrometry. After comparing the obtained data (Additional file 1: See Supporting information S1) with those reported in the literature, the compounds were identified as 3-acetoxy-11-oxo-urs-12-ene (1), *p*-coumaric acid (2), citric acid trimethyl ester (3), resveratrol (4), resveratrol *β-*_D_*-*glucopyranoside (5) and strictosamide (6).

### Cell cultures

The cell lines used in the present work, their origins and their treatments were previously reported [18, 19]. They include drug-sensitive leukemia CCRF-CEM cells, its multidrug-resistant subline CEM/ADR5000 cells [3, 20, 21], breast cancer MDA-MB-231-pcDNA3 cells, its resistant subline MDA-MB-231-*BCRP* clone 23) cells [22], colon HCT116 (*p53*^*+/+*^) cancer cells, its knockout clones HCT116 (*p53*^*−/−*^), glioblastoma U87MG cells, its resistant subline U87MG.Δ*EGFR* cells and normal AML12 hepatocytes [14, 15, 19, 23]. The CCRF-CEM and CEM/ADR5000 leukemia cells were maintained in RPMI 1640 medium (Invitrogen) supplemented with 10 % fetal calf serum in a humidified 5 % CO_2_ atm at 37 °C. Breast cancer cells, transduced with control vector (MDA-MB-231-pcDNA3) or with cDNA for the breast cancer resistance protein *BCRP* (MDA-MB-231-*BCRP* clone 23), were maintained under standard conditions as described above for CCRF-CEM cells. Human wild-type HCT116 (*p53*^*+/+*^) colon cancer cells as well as knockout clones HCT116 (*p53*^*−/−*^) derived by homologous recombination were a generous gift from Dr. B. Vogelstein and H. Hermeking (Howard Hughes Medical Institute, Baltimore, MD). Human glioblastoma multiforme U87MG cells (non-transduced) and U87MG cell line transduced with an expression vector harboring an epidermal growth factor receptor (*EGFR*) gene with a genomic deletion of exons 2 through 7 (U87MG.Δ*EGFR*) were kindly provided by Dr. W. K. Cavenee (Ludwig Institute for Cancer Research, San Diego, CA). MDA-MB-231-*BCRP,* U87MG.Δ*EGFR* and HCT116 *(p53*^*−/−*^*)* were maintained in DMEM medium containing 10 % FBS (Invitrogen) and 1 % penicillin (100 U/mL)-streptomycin (100 μg/mL) (Invitrogen) and were continuously treated with 800 ng/mL and 400 μg/mL geneticin, respectively. Normal AML12 heptocytes were obtained from the American Type Culture Collection (ATCC, USA). The above medium without geneticin was used to maintain MDA-MB-231, U87MG, HCT116 (*p53*^*+/+*^) and AML12 cell lines. The cells were passaged twice weekly. All experiments were performed with cells in the logarithmic growth phase.

### Resazurin reduction assay

The cytotoxicity of the tested samples was performed by resazurin reduction assay as we previously described [14, 15, 18, 19, 24, 25]. Briefly, adherent cells at 1 × 10^4^ cells were allowed to attach overnight and were then treated with different concentrations of the studied samples. For suspension cells, aliquots of 2 × 10^4^ cells per well were seeded in 96-well-plates in a final volume of 200 μL. Extracts and compounds were prior diluted in DMSO and tested in a final concentration below 0.1 % (A final concentration of 0.1 % DMSO was used as negative control and did not show any effect on cell growth). After 72 h incubation and resazurin (Sigma-Aldrich, Schnelldorf, Germany) staining, fluorescence was measured on an Infinite M2000 Pro™ plate reader (Tecan, Crailsheim, Germany) using an excitation wavelength of 544 nm and an emission wavelength of 590 nm. Each assay was done at least two times, with six replicates each. IC_50_ values represent the sample concentration required to inhibit 50 % of cell proliferation and were calculated from a calibration curve by linear regression using Microsoft Excel.

## Results and discussion

Compounds were identified as 3-acetoxy-11-oxo-urs-12-ene C_32_H_50_O_3_ (1; m.p. 282.1-283.4 °C; *m*/*z*: 482.4; purity: 90 %)[26], *p*-coumaric acid C_9_H_8_O_3_ (2; *m*/*z*: 164.0; purity: 97 %)[27], citric acid trimethyl ester C_9_H_14_O_7_ (3; *m*/*z*: 234.0; purity: 97 %)[28], resveratrol C_14_H_12_O_3_ (4; *m*/*z*: 228.1; purity: 98 %)[29], resveratrol *β-*_D_*-*glucopyranoside C_20_H_22_O_8_ (5; *m*/*z*: 390.1; purity: 95 %)[30], and strictosamide C_26_H_30_N_2_O_8_ (6; *m*/*z*: 498.2; purity: 96 %) [31]. The irido-indole alkaloid strictosamide (6, 35 mg) was the major constituent of the bark extract. This is in accordance with previous studies reporting 6 as the main compound isolated from the bark methanolic extract of *Nauclea pobeguinii* harvested in Democratic Republic of Congo [32]. However, Xu et al. [32] also identified several other alkaloids as minor constituents of the bark extract, such as naucleidinic acid and 19-*O*-methyl-3,14-dihydroangustoline, naucleidinal, magniflorine, naucleofficine D, 3,14-dihydroangustoline, strictosidine, desoxycordifoline, 3*a*,5*a*-tetrahydrodeoxycordifoline lactam, and a phenol kelampayoside A. These compounds were not isolated in our sample from Cameroon. In addition, compounds 1–5 reported in this study were also not found in the plant harvested in Congo. This could either be due to the isolation techniques used or to the environmental variation that influences the concentration of the minor constituents synthesized by the *Nauclea pobeguinii*. strictosamide (6) was obtained as the major constituent isolated from the methanolic extract in both cases.

**Fig. 2:**
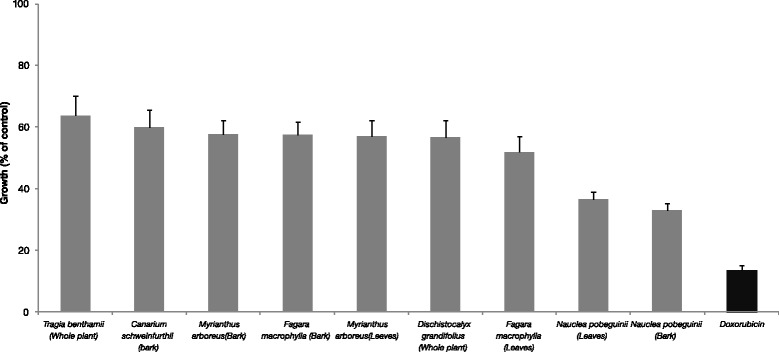
Growth percentage (%) of CCRF-CEM leukemia cells treated with plant extracts and doxorubicin. Samples were tested at a single concentration of 40 mg/mL for crude extracts and of 10 μM for doxorubicin

A preliminary cytotoxicity study was first carried out on leukemia CCRF-CEM cells with crude extracts at a single concentration of 40 μg/mL for crude extracts. Doxorubicin (10 μM) served as positive control. According to the National Cancer Institute (USA), 30 μg/mL is the upper IC_50_ limit considered promising for purification of a crude extract [33]. We tested a slightly higher concentration (of 40 μg/mL) in our preliminary assay. The results depicted in Fig. 2 show that more than 50 % growth was obtained if CCRF-CEM cells were treated with the methanol extracts from *Tragia benthamii* (63.7 %), *Canarium schweinfurthii* (59.96 %), *Myrianthus arboreus* (57.8 %), *Dischistocalyx grandifolius* (56.8 %) and *Fagara macrophylla* leaves (52.0 %)*.* Only the crude extracts from the leaves (36.6 %) and bark (33.0 %) of *Nauclea pobeguinii* as well as doxorubicin (13.6 %) were able to induce more than 50 % inhibition of CCRF-CEM leukemia cell growth (Fig. 2). The IC_50_ values of extracts (bark and leaves) from *Nauclea pobeguinii* were further determined on eight cancer cell lines and values below 30 μg/mL were obtained both extracts towards two out of eight and five out of eight cell lines, respectively (Table 2). The lowest IC_50_ value of 8.70 μg/mL was obtained with the bark extract against HCT116 *(p53*^*−/−*^*)* cells. Consequently, this extract was subjected to purification from which six compounds (1–6) were obtained. Compounds 4 and 6 displayed selective activities on the studied cancer cell lines. IC_50_ values below 100 μM were recorded with compound 5 on all eight tested cancer cell lines. IC_50_ values below 175.36 μM and 80.29 μM (for 4 and 6 respectively) were also obtained against seven out of eight cell lines for 4 and two out of eight cell lines for 6. The IC_50_ values ranged from 25.08 μM (towards CCRF-CEM cells) to 97.64 (towards MDA-MB231 cells) for compound 5 and from 0.20 μM (against CCRF-CEM cells) and 195.12 μM (against CEM/ADR5000 cells) for doxorubicin. No IC_50_ values were obtainable, if compound 1 was tested at up to 82.92 μM. The same is true for compounds 2 (>243.90 μM) and 3 (>170.94 μM). The best compounds (4 and 5) were less toxic towards AML12 normal hepatocytes than against cancer cells. The IC_50_ threshold values of 20 μg/mL for crude extracts as well as 4 μg/mL or 10 μM for compounds [34, 35] after 48 and 72 h incubation have been set by the United States National Cancer Institute (USNCI) to identify good cytotoxic phytochemicals. None of the tested compounds displayed IC_50_ values below 10 μM. However, the bark extract of *Nauclea pobeguinii* (IC_50_ value below 20 μg/mL on four out of eight tested cancer cell lines) could be considered as a promising candidate to fight cancers. The best isolated compounds (4–6) displayed rather moderate activities, suggesting possible synergistic effects of constituents in the crude extract.

**Table 2:** Cytotoxicity of extracts and compounds from *Nauclea pobeguinii* towards sensitive and drug-resistant cancer cell lines and normal cells as determined by the resazurin assay

Cell lines	Tested samples, IC_50_ values and degrees of resistance (in bracket)								
	Crude extracts and IC_50_ values (μg/mL)		IC_50_ values of compounds (μM)						IC_50_ values of doxorubicin (μM)
	NPB	NPL	1	2	3	4	5	6	
CCRF-CEM	14.62 ± 1.23	25.84 ± 2.16	>82.92	>243.90	>170.94	28.15 ± 1.32	25.08 ± 2.48	77.10 ± 5.87	0.20 ± 0.06
CEM/ADR5000	11.56 ± 0.85 (0.80)	25.55 ± 1.63 (0.99)	>82.92 (n.a)	>243.90 (n.a)	>170.94 (n.a)	57.43 ± 3.67 (2.04)	39.87 ± 2.91 (1.59)	>80.29 (>1.04)	195.12 ± 14.30 (975.60)
MDA-MB231	37.00 ± 2.91	64.75 ± 5.08	>82.92	>243.90	>170.94	19.90 ± 1.45	97.64 ± 7.60	>80.29	1.10 ± 0.28
MDA-MB231*/BCRP*	19.79 ± 2.13 (0.53)	59.55 ± 4.21 (0.92)	>82.92 (n.a)	>243.90 (n.a)	>170.94 (n.a)	22.93 ± 1.64 (1.15)	95.59 ± 8.17 (0.98)	78.26 ± 6.22 (<0.97)	7.83 ± 0.47 (7.12)
HCT116 *(p53* ^*+/+*^ *)*	16.19 ± 1.16	32.78 ± 2.67	>82.92	>243.90	>170.94	73.65 ± 5.67	63.77 ± 4.61	>80.29	1.41 ± 0.29
HCT116 *(p53* ^*−/−*^ *)*	8.70 ± 0.68 (0.54)	19.39 ± 1.34 (0.59)	>82.92 (n.a)	>243.90 (n.a)	>170.94 (n.a)	>175.36 (>2.38)	47.03 ± 4.94 (0.74)	>80.29 (n.a)	4.06 ± 0.07 (2.88)
U87MG	69.44 ± 4.34	>40	>82.92	>243.90	>170.94	76.59 ± 4.92	55.64 ± 3.73	>80.29	1.06 ± 0.15
U87MG*.ΔEGFR*	32.78 ± 2.79 (0.47)	63.90 ± 4.77 (>1.60)	>82.92 (n.a)	>243.90 (n.a)	>170.94 (n.a)	28.45 ± 2.06 (0.37)	47.59 ± 3.29 (0.86)	>80.29 (n.a)	6.11 ± 0.57(5.76)
AML12	>40	>40	>82.92	>243.90	>170.94	>175.36	>102.56	>80.29	>73.59

(*): The degree of resistance was determined as the ratio of IC_50_ value in the resistant divided by the IC_50_ in the sensitive cell line; NPB: extract from the bark of *Nauclea pobeguinii*; NPL: extract from the leaves of *Nauclea pobeguinii*; Compounds isolated from NPB [**1:** 3-acetoxy-11-oxo-urs-12-ene; **2:** *p*-coumaric acid; **3:** citric acid trimethyl ester; **4:** resveratrol; **5:** resveratrol *β-*_D_ *-*glucopyranoside; **6:** strictosamide]; n.a.: not applicable. IC_50_ value are mean ± SD of at least two experiments with six replicates each

The development of MDR in cancer cells either through ABC transporters [36], the epidermal growth factor receptor (EGFR) [2–4], or the tumor suppressor p53 gene [5] represents a major hurdle in chemotherapy. The discovery of new compounds with activity against MDR is hence very important in the ongoing fight against malignancies. In the present study, we used cell lines possessing all these resistance mechanisms to investigate multi-factorial drug resistance. The degrees of resistance were determined as the ratio of IC_50_ value of the resistant cell line to that of the corresponding parental sensitive counterpart (Table 2). Compared to their corresponding sensitive cell lines, collateral sensitivity in resistant cells (hypersensitivity) was observed in P-glycoprotein-overexpressing CEM/ADR5000 cells (degree of resistance 0.80-fold), BCRP-overexpressing MDA-MB-231-*BCRP* cells (0.53-fold), p53 HCT116 (*p53*^*−/−*^) knockout cells (<0.54-fold) and epidermal growth factor receptor-overexpressing U87MG.Δ*EGFR* cells (0.47-fold) to the bark extract of *N. pobeguinii.* Collateral sensitivity was also found in HCT116 (*p53*^*−/−*^) cells (0.74-fold) and U87MG.Δ*EGFR* cells (0.86-fold) to compound 5.

The obtained data indicates that the stilbene resveratrol glucoside 5 and its aglycon 4 displayed slightly different degrees of activity on the cancer cell lines studied. This shows that glucosylation may positively (especially in leukemia cells) influence the cytotoxic activity. In fact, resveratrol glucoside 5 was more active than its aglcon 4 on the two tested leukemia cells. However, in carcinoma cell lines, the cytotoxicity of compounds 4 and 5 varied from one cell lines to other, none of two being more active than other one in all the solid cancer cell lines tested. To the best of our knowledge, this phytochemical and cytotoxicity study of the crude bark and leaf extracts as well as compounds 1–3 and 5 of *Nauclea pobeguinii* towards multifactorial drug resistant cancer lines is being reported here for the first time. Though strictosamide (6) is known to be the main constituent [32] of this plant, the present study suprisingly showed that it was not the most cytotoxic component of the extracts against the studied cancer cell lines. The best activities were reported with stilbenes namely resveratrol (4) and it glycoside resveratrol *β-*_D_*-*glucopyranoside (5). Compound 4 is a well known cytotoxic agent [37–39]. It is reported to suppress the proliferation of SKBR-3 breast cancer cells by inhibiting fatty acid synthase signaling pathway [38]. Compound 4 also alleviates the PI3K/Akt/mTOR signaling in breast cancer SKBR-3cells by down-regulation of Akt phosphorylation and up-regulation of PTEN expression [38]. Besides, compound 4 reportedly reverses MDR of the MCF-7/DOX breast cancer cells [39]. In the present study, compound 4 was most effective (IC_50_ < 23 μM) against MDA-MB231 breast adenocarcinoma cells and their drug-resistant, MDA-MB231*/BCRP*. These data are in accordance with previous reports and consolidates the potential cytotoxicity of 4 against breast cancer cells.

## Conclusion

In conclusion, we demonstrate the cytotoxic potential of six Cameroonian medicinal plants, *Canarium schweinfurthii*, *Dischistocalyx grandifolius*, *Tragia benthamii*, *Fagara macrophylla*, *Myrianthus arboreus* and *Nauclea pobeguinii*. We also demonstrated the cytotoxic potential of leaves and bark of *Nauclea pobeguinii* against sensitive and MDR cancer cell lines. We further identified resveratrol and its *β-*glucoside as major cytotoxic constituents of the bark of *Nauclea pobeguinii*. The bark and leaves extracts of *Nauclea pobeguinii* are potential cytotoxic botanicals that deserves more investigations to develop novel cytotoxic phytomedicines against drug-resistant cancers.

## Additional files

Additional file 1:
**Supporting information S1.** Data on compounds isolated from *Nauclea pobeguiinii (DOC 860 kb)*
