# Supplementary material for: Cytotoxicity of selected Cameroonian medicinal plants and Nauclea pobeguinii towards multi-factorial drug-resistant cancer cells
Source: BMC Complement Altern Med. 2015 Sep 4;15:309. doi: 10.1186/s12906-015-0841-y (PMC4559964; doi:10.1186/s12906-015-0841-y)
Supplement: Additional file 1: — Supporting information S1. Data on compounds isolated from Nauclea pobeguiinii (DOC 860 kb) [file 12906_2015_841_MOESM1_ESM.doc]

**Cytotoxicity of Selected Cameroonian Medicinal Plant and *Nauclea pobeguinii* Towards Multi-Factorial Drug Resistant Cancer Cells**

Victor Kuete^1,2^, Louis P. Sandjo^3^, Armelle T. Mbaveng^2^, Jackson A. Seukep^2^, Armelle T. Mbaveng^2^, Bonaventure Ngadjui^4^, Thomas Efferth^1*^

*^1^Department of Pharmaceutical Biology, Institute of Pharmacy and Biochemistry, University of Mainz, Staudinger Weg 5, 55128 Mainz, Germany;*

*^2^Department of Biochemistry, Faculty of Science, University of Dschang, Cameroon;*

*^3^Department of Pharmaceutical Sciences, CCS, Universidade Federal de Santa Catarina, Florianópolis, 88040-900, SC, Brazil;*

*^4^Department of Organic Chemistry, Faculty of Science, University of Yaoundé 1, Cameroon.*

*****Corresponding author:**

*Tel: (+49) 6131-3925751; Fax: (+49) 49-6131-3923752; E-mail:* [*efferth@uni-mainz.de*](mailto:efferth@uni-mainz.de)*; 55128 Mainz, Germany (Prof. Dr. Thomas Efferth)*

**Running title**: Cytotoxic constituents of *Nauclea pobeguinii*

**S1. Data on compounds isolated from *Nauclea pobeguiinii***

*3-acetoxy-11-oxo-urs-12-ene* (**1**). Colorless powder, m.p. [282.1-283.4] ^o^C; LR-EI-MS *m*/*z*: 482.4 [C_32_H_50_O_3_]; Rf 2/5 (Hex/EA 9:1); Degree of purity 90 %; ^1^H-NMR (CDCl_3_, 100 MHz): δ 0.84 (overlapped with H-19, H-5), 0.94 (s, H-23), 0.95 (s, H-24), 1.32 (s, H-25), 1.16 (s, H-26), 1.35 (s, H-27), 0.80 (s, H-28), 0.77 (d, J = 6.4 Hz, H-29), 0.89 (d, J = 6.4 Hz, H-30), 1.48 (overlapped with H-6, H-18), 2.08 (s, H-2’), 2.47 (s, H-9), 4.74 (dd, J = 4.8, 11.8 Hz, H-3), 5.76 (s, H-12). ^13^C-NMR (CDCl_3_, 100 MHz): δ 39.4 (C-1), 24.4 (C-2), 80.8 (C-3), 38.6 (C-4), 55.4 (C-5), 18.1 (C-6), 33.2 (C-7), 45.6 (C-8), 61.9 (C-9), 37.6 (C-10), 199.4 (C-11), 131.0 (C-12), 165.1 (C-13), 44.3 (C-14), 28.0 (C-15), 27.8 (C-16), 34.4 (C-17), 59.3 (C-18), 39.8 (C-19), 39.6 (C-20), 31.4 (C-21), 41.4 (C-22), 28.6 (C-23), 17.4 (C-24), 17.3 (C-25), 19.0 (C-26), 21.1 (C-27), 29.3 (C-28), 17.8 (C-29), 21.6 (C-30), 171.1 (C-1’), 21.6 (C-2’) [1].


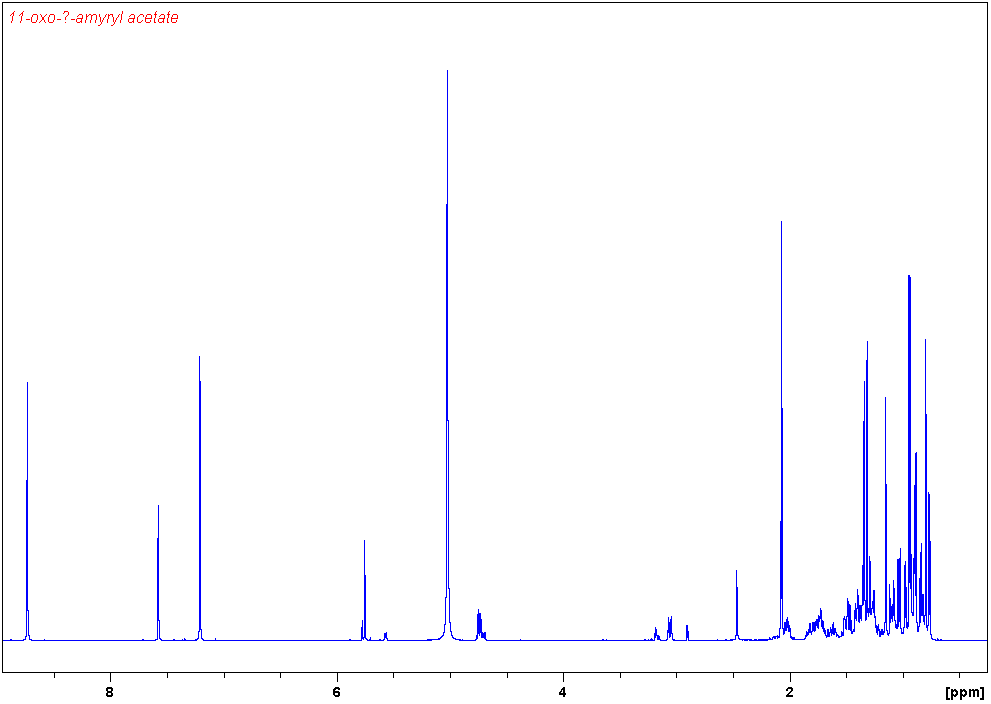


^1^H NMR spectrum of compound **1** in pyridine-*d_5_*


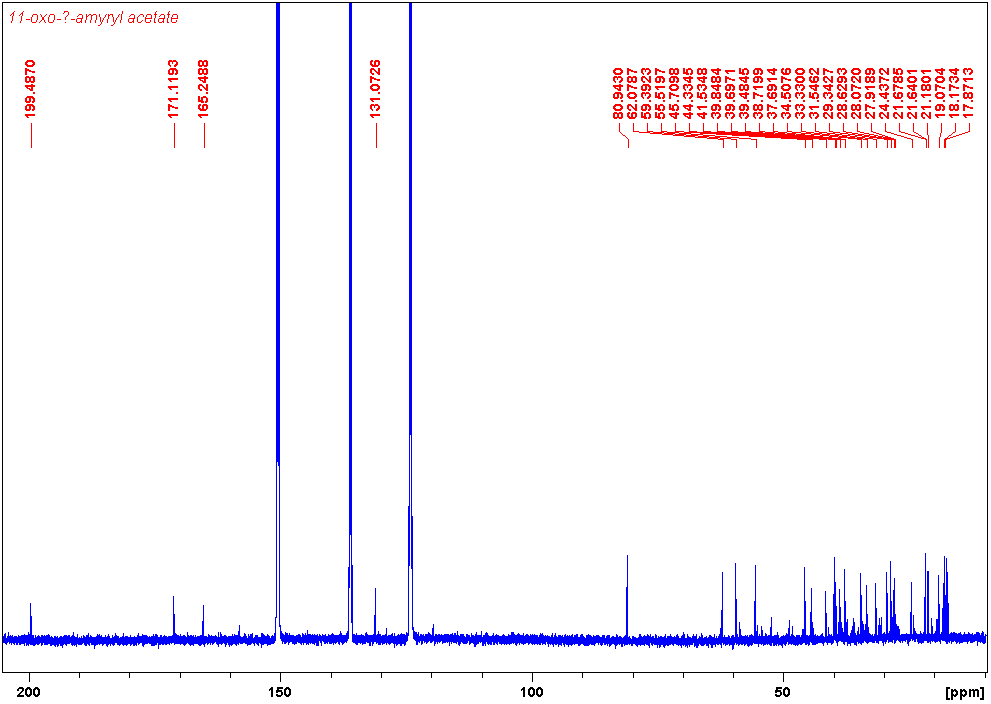


^13^C NMR spectrum of compound **1** in pyridine-*d_5_*

*p*-*Coumaric acid* (**2**). Brown oil, LR-EI-MS *m*/*z*: 164.0 [C_9_H_8_O_3_], Rf 1.95/5 (Hex/EA 9:1), Degree of purity 97%; ^1^H- NMR (C_5_D_5_N, 400 MHz): δ 6.87 (d, J = 15.9 Hz, H-2), 8.14 (d, J = 15.9 Hz, H-3), 7.66 (d, J = 8.6 Hz, H-5 and H-9), 7.18 (d, J = 8.6 Hz, H-6 and H-8) ^13^C-NMR (C_5_D_5_N, 100 MHz): δ 170.1 (C-1), 117.5 (C-2), 145.0 (C-3), 126.9 (C-4), 131.0 (C-5 and C-9), 117.3 (C-6 and C-8), 161.6 (C-7) [2].


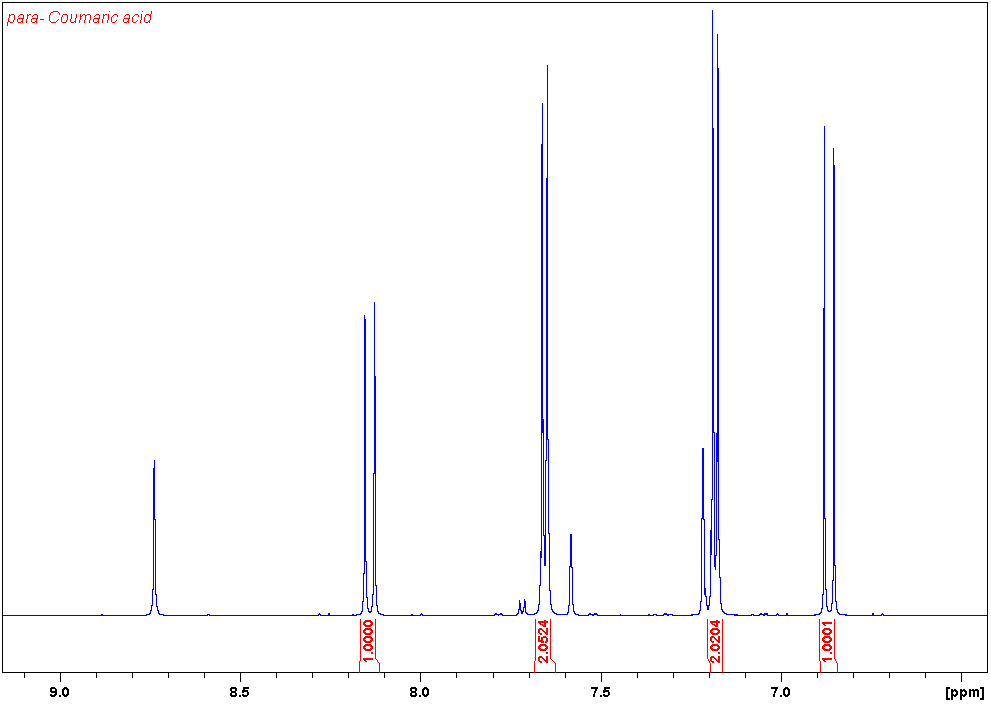


^1^H NMR spectrum of compound **2** in pyridine-*d_5_*


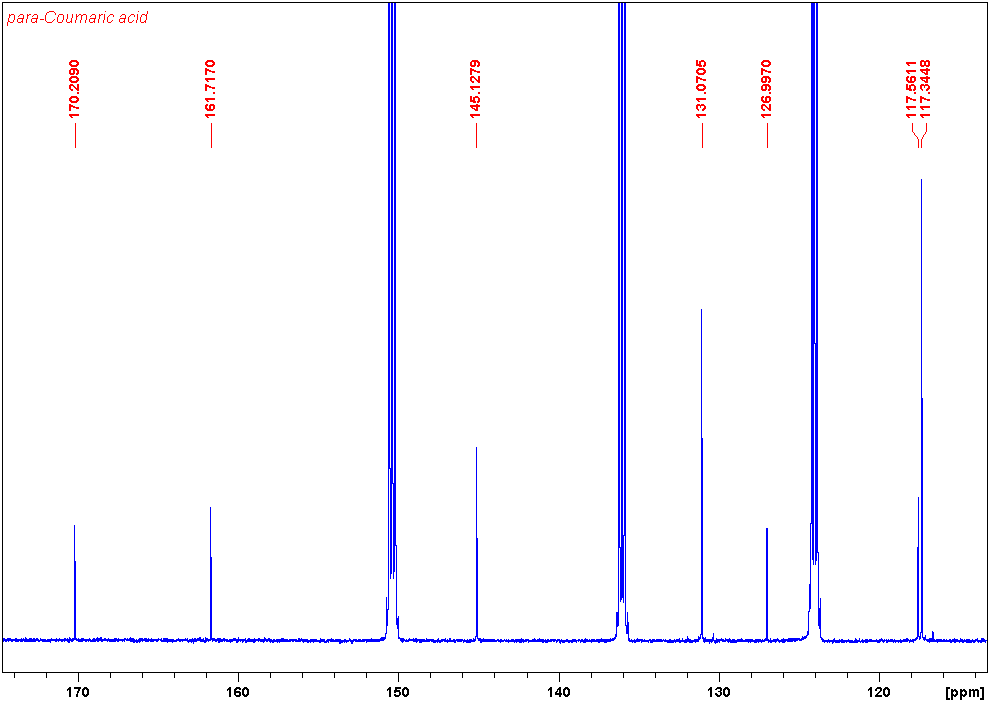


^13^C NMR spectrum of compound **2** in pyridine-*d_5_*

*Citric acid trimethyl ester* (**3**) colorless sticky gum, LR-EI-MS *m*/*z*: 234.0 [C_9_H_14_O_7_], Rf 2.5/5 (DCM), Degree of purity 97 %; ^1^H- NMR (CDCl_3_, 400 MHz): δ 2.81 (d, J = 15.7 Hz, H-2a and H-4a), 2.91 (d, J = 15.7 Hz, H-2b and H-4b), 3.69 (s, MeO-1 and MeO-5), 3.84 (s, MeO-6), 4.13 (s, OH-3). ^13^C-NMR (CDCl_3_, 100 MHz): δ 170.4 (C-1 and C-5), 43.2 (C-2 and C-4), 73.4 (C-3), 174.0 (C-6), 52.5 (MeO-1 and MeO-5), 53.4 (MeO-6)[3].


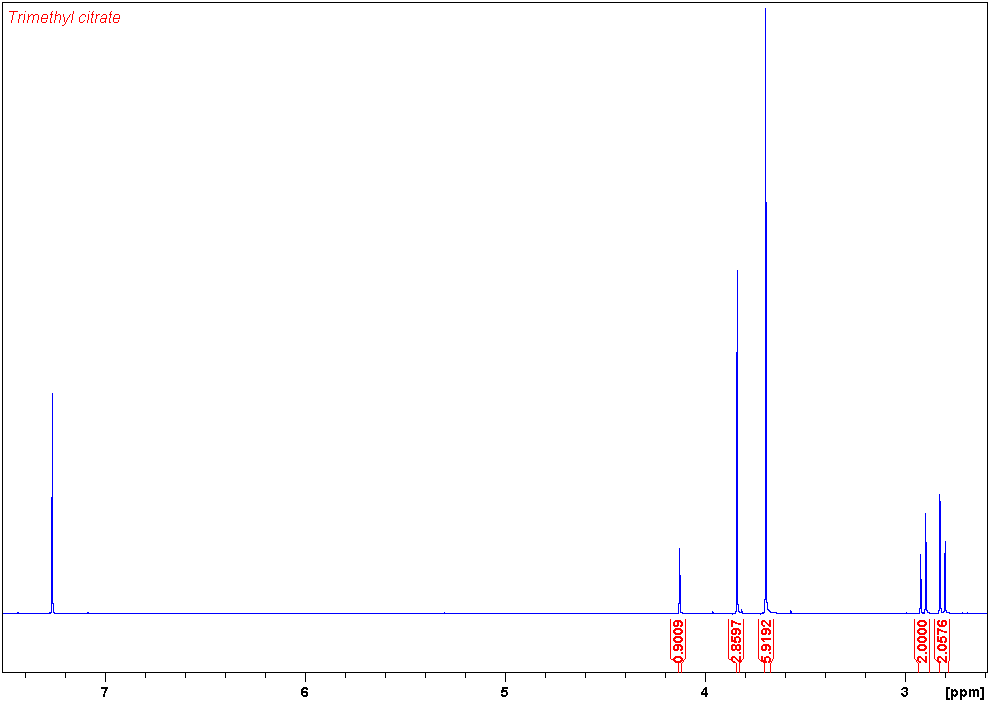


^1^H NMR spectrum of compound **3** in CDCl_3_


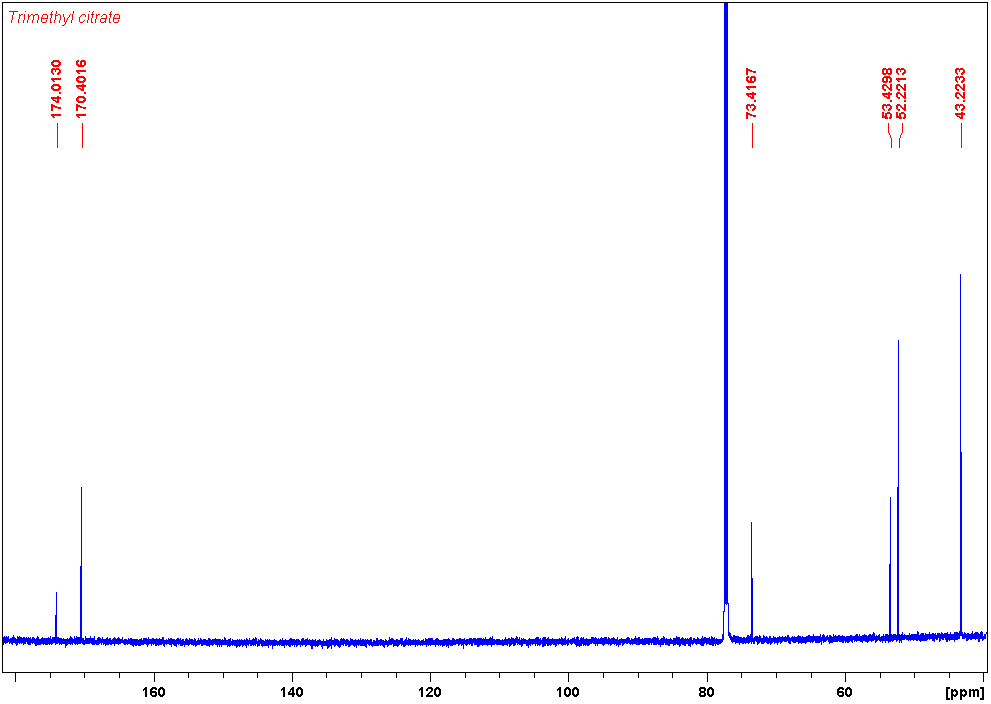


^13^C NMR spectrum of compound **3** in CDCl_3_

*Resveratrol* (**4**) brownish solid, mp 258.5–260.1 °C, LR-EI-MS *m*/*z*: 228.1 [C_14_H_12_O_3_], Rf 3/5 (DCM-MeOH 97:3), Degree of purity 98 %;  ^1^H- NMR (C_5_D_5_N, 400 MHz): δ 7.19 ( overlapped with H-10 and H-14, H-2 and H-6), 7.58 (d, J = 8.6 Hz, H-3 and H-5), 7.40 (d, J = 16.3 Hz, H-7), 7.30 (d, J = 16.3 Hz, H-8), 7.20 (overlapped with H-2 and H-6, H-10 and H-14), 7.00 (t, J = 2.2 Hz, H-12), 11.5 (s, OH-11 and OH-13), 11.8 (s, OH-1) ^13^C-NMR (C_5_D_5_N, 100 MHz): δ 159.4 (C-1), 117.0 (C-2 and C-6), 129.0 (C-3 and C-5), 129.6 (C-4), 129.4 (C-7), 127.3 (C-8), 141.3 (C-9), 106.3 (C-10 and C-14), 161.0 (C-11 and C-13), 103.9 (C-12)[4].


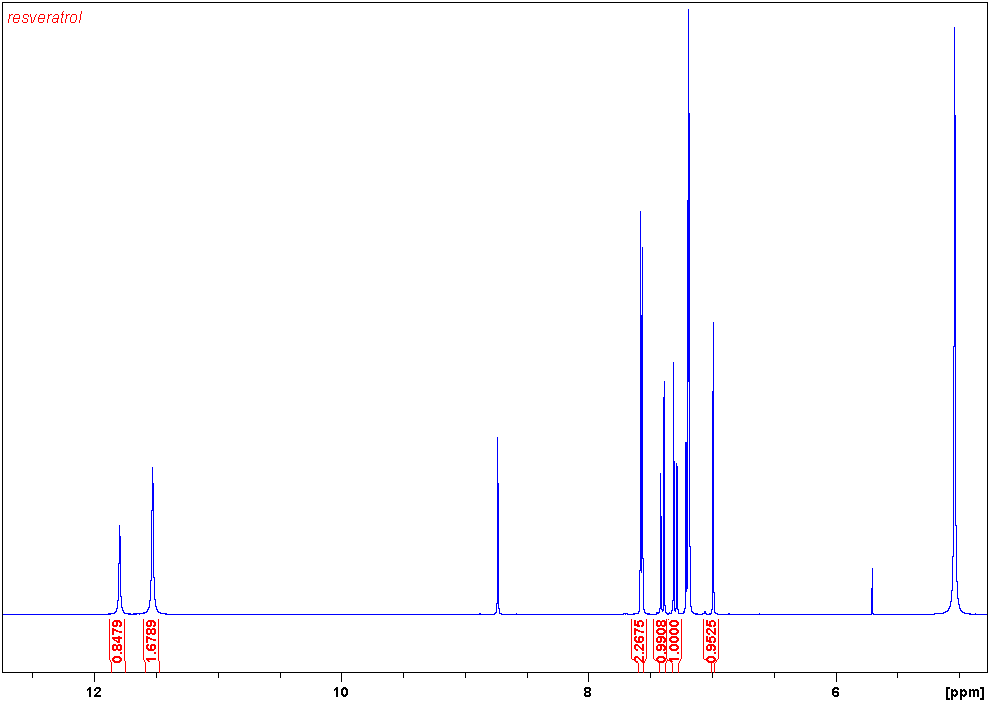


^1^H NMR spectrum of compound **4** in pyridine-*d_5_*


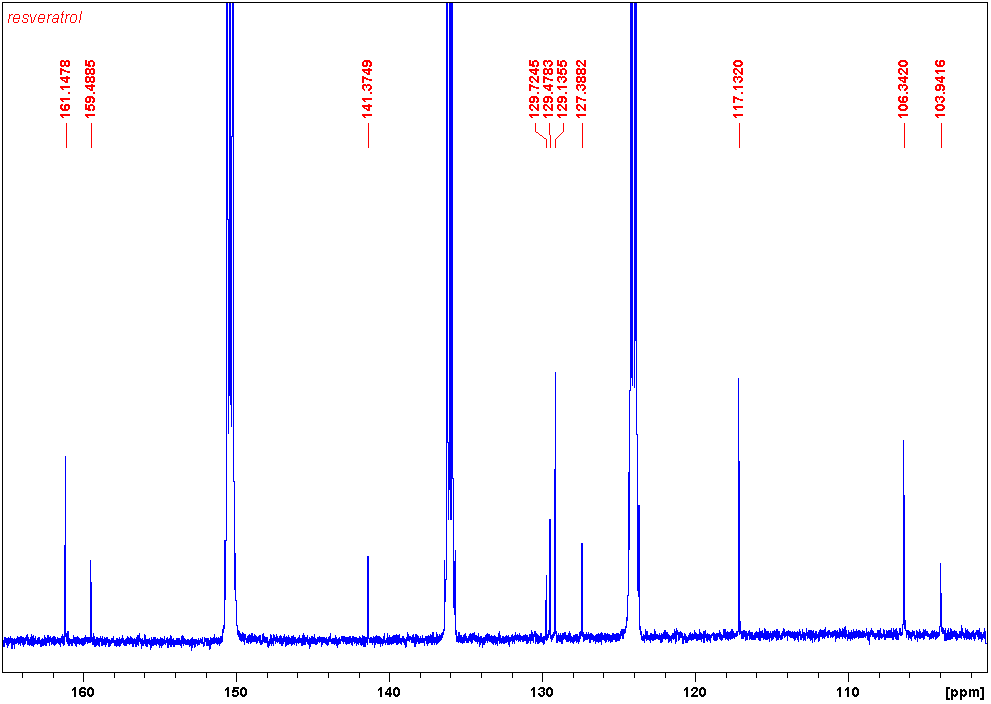


^13^C NMR spectrum of compound **4** in pyridine-*d_5_*

*resveratrol β-D-glucopyranoside* (**5**) colorless solid, m.p. 225–226.9 °C, LR-EI-MS *m*/*z*: 390.1 [C_20_H_22_O_8_], Rf 3/5 (DCM-MeOH 95:5), Degree of purity 95 %;  ^1^H- NMR (C_5_D_5_N, 400 MHz): δ 7.18 (d, J = 8.6 Hz, H-2 and H-6), 7.58 (d, J = 8.6 Hz, H-3 and H-5), 7.43 (d, J = 16.1 Hz, H-7), 7.22 (d, J = 16.1 Hz, H-8), 7.21 (d, J = 2.4 Hz, H-10), 7.15 (t, J = 2.4 Hz, H-12), 7.43 (overlapped with the solvent signal, H-14), 5.74 (d, J = 7.0 Hz, H-1’), 4.36-4.40 (m, H-2, H-4 and H-5), 4.10 (m, H-3), 4.52 (dd, J = 2.4, 12.1 Hz, H-6’), 4.41 (dd, J = 5.4, 12.1 Hz, H-6’), 11.8 (s, OH-1 and OH-11). ^13^C-NMR (C_5_D_5_N, 100 MHz): δ 159.5 (C-1), 117.0 (C-2 and C-6), 129.1 (C-3 and C-5), 129.5 (C-4), 130.0 (C-7), 126.7 (C-8), 141.2 (C-9), 109.1 (C-10), 160.8 (C-11 and C-13), 104.4 (C-12), 106.3 (C-14), 102.9 (C-1’), 75.5 (C-2’), 79.4 (C-3’), 71.6 (C-4’), 79.0 (C-5’), 62.7 (C-6’)[5].


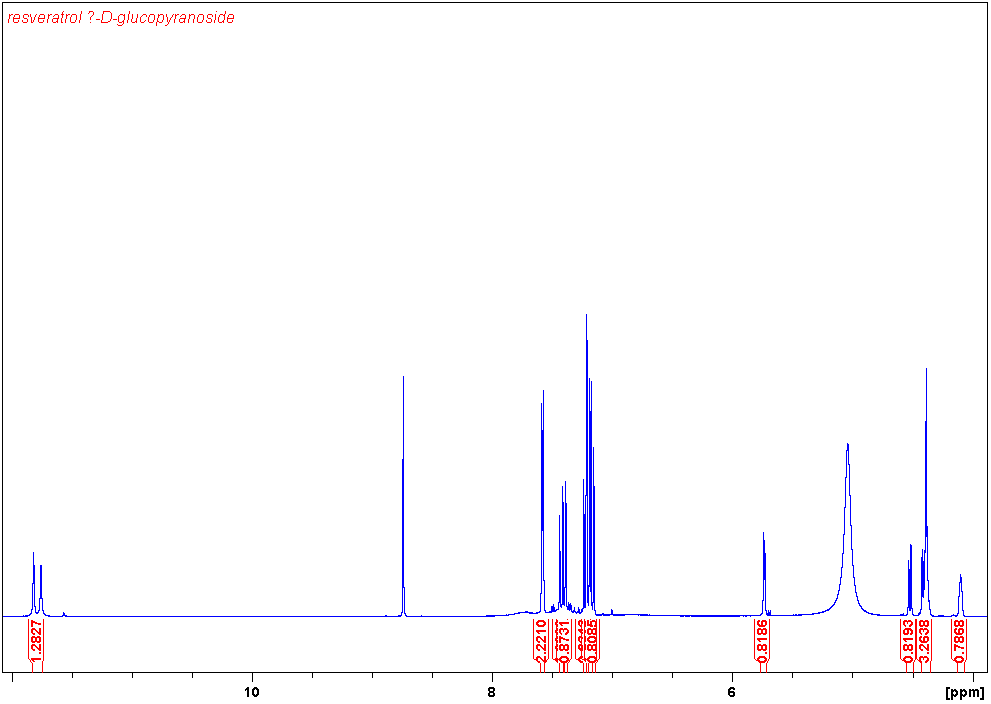


^1^H NMR spectrum of compound **5** in pyridine-*d_5_*


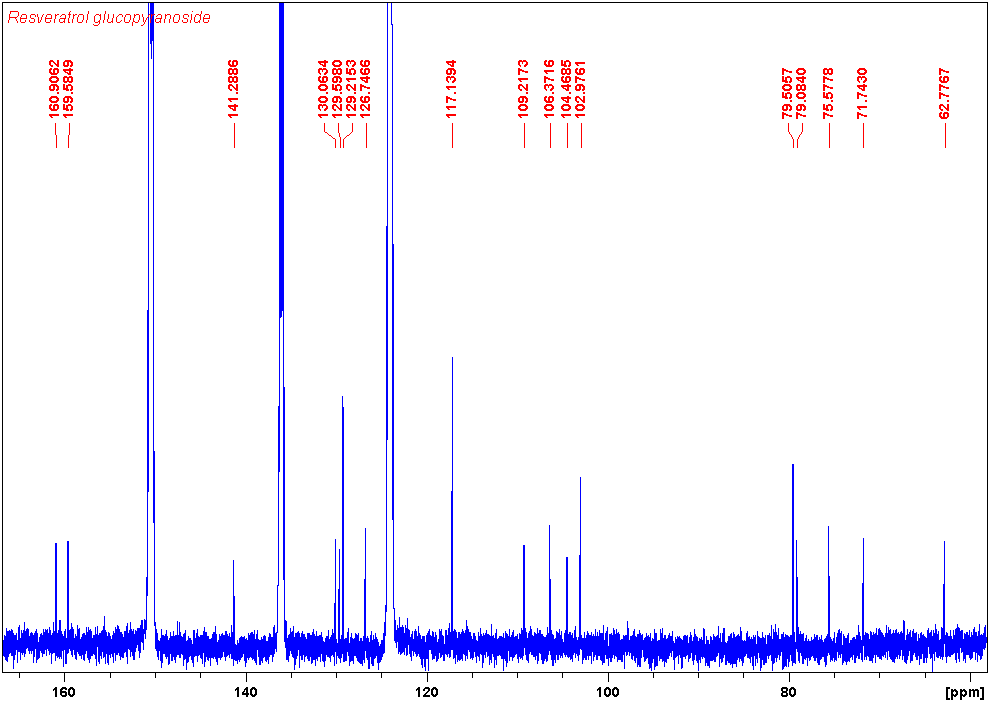


^13^C NMR spectrum of compound **5** in pyridine-*d_5_*

Strictosamide (**6**) yellow solid, m.p. 225–226.9 °C, LR-EI-MS *m*/*z*: 498.2 [C_26_H_30_N_2_O_8_], Rf 1.8/5 (DCM-MeOH 9:1), Degree of purity 96 %; ^1^H-NMR (CD_3_OD, 400 MHz): δ 5.04 (m, H-3), 3.15 (m, H-5a), 4.94 (m, H-5b), 2.76 (m, H-6a), 2.99 (m, H-6b), 7.35 (br d, J = 7.3 Hz, H-9), 6.98 (*pseudo*-t, J = 7.3 Hz, H-10), 7.08 (dd, J = 7.3, 7.7 Hz, H-11), 7.32 (d, J = 7.7 Hz, H-12), 2.02 (m, H-14a), 2.65 (m, H-14b), 2.46 (m, H-15), 7.38 (s, H-17), 5.30 (dd, J = 1.8, 10.0 Hz, H-18a), 5.36 (dd, J = 1.8, 17.5 Hz, H-18b), 5.67 (m, H-19), 2.68 (m, H-20), 5.39 (d, J = 1.9 Hz, H-21), 4.57 (d, J = 7.7 Hz, H-1’), 3.19-3.22 (m, H-2’, H-3’, H-5’), 3.61 (*pseudo*-t, J = 9.0 Hz, H-4’), 3.66 (dd, J = 2.4, 12.2 Hz, H-6’), 3.84 (dd, J = 5.4, 12.2 Hz, H-6’). ^13^C-NMR (C_5_D_5_N, 100 MHz): δ 167.1 (C-22), 149.1 (C-17), 135.1 (C-13), 137.5 (C-2), 134.4 (C-19), 128.6 (C-8), 122.6 (C-10), 120.6 (C-18), 120.2 (C-11), 118.9 (C-9), 112.3 (C-12), 110.0 (C-7), 109.9 (C-16), 99.0 (C-21), 55.0 (C-3), 44.8 (C-5), 44.9 (C-20), 23.1 (C-14), 24.9 (C-15), 27.1 (C-6). 101.5 (C-1’), 74.1 (CH, C-2’), 78.0 (C-3’), 71.4 (C-4’), 78.2 (C-5’), 62.6 (C-6’) [6].


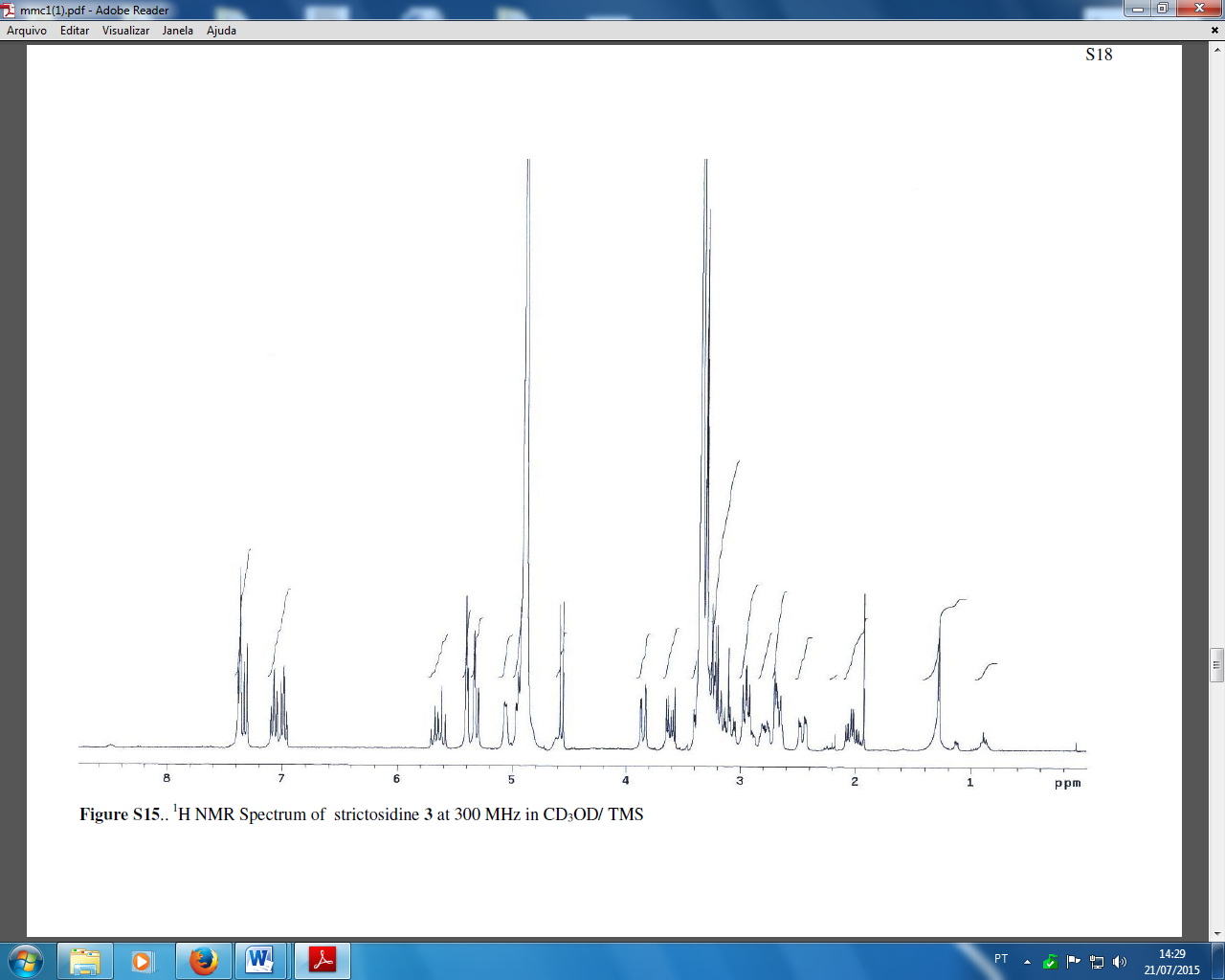


^1^H NMR spectrum of compound **6** in CD_3_OD


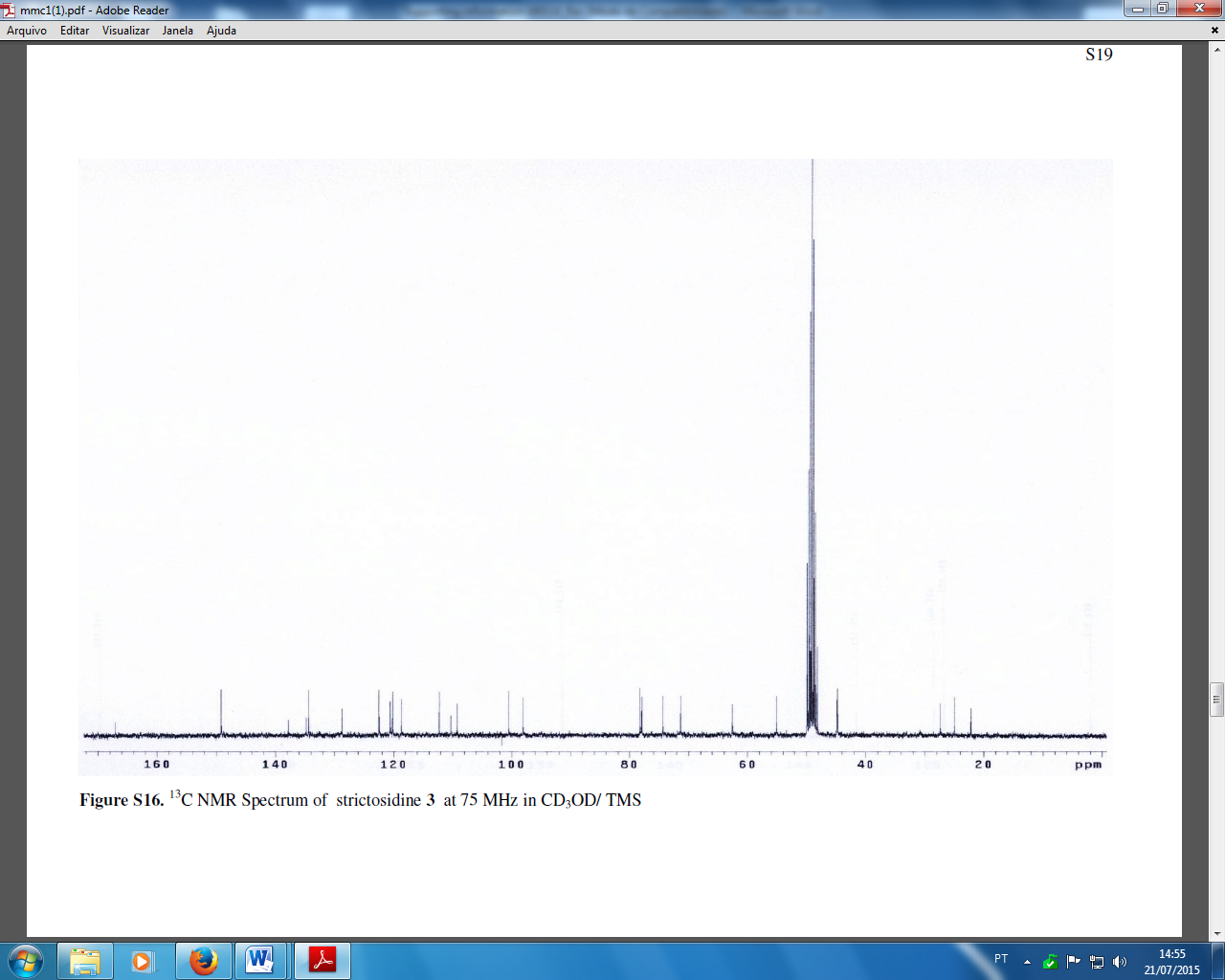


^13^C NMR spectrum of compound **6** in CD_3_OD

**References**

1. Ogawa S, Wakatsuki Y, Makino M, Fujimoto Y, Yasukawa K, Kikuchi T, Ukiya M, Akihisa T, Iida T. **Oxyfunctionalization of unactivated C-H bonds in triterpenoids with tert-butylhydroperoxide catalyzed by meso-5,10,15,20-tetramesitylporphyrinate osmium(II) carbonyl complex.** Chemi Physics Lipids. 2010; **163**:165-171.

2. Huang Y, Zeng W, Li G, Liu G, Zhao D, Wang J, YL Z. **Characterization of a new sesquiterpene and antifungal activities of chemical constituents from *Dryopteris fragrans* (L.) Schott**. Molecules 2014; **19**:507-513.

3. Choi J, Lee D: **A new citryl glycoside from *Gastrodia elata* and its inhibitory activity on GABA transaminase**. Chem Pharm Bull. 2006; **54**:1720-1721.

4. Aydin T, Cakir A, Kazaz C, Bayrak N, Bayir Y, Taskesenligil Y. **Insecticidal metabolites from the rhizomes of *Veratrum album* against adults of Colorado potato beetle, *Leptinotarsa decemlineata***. Chem Biodivers. 2014; **11**:1192-1204.

5. Wei X, Yang S, Liang N, Hu D, Jin L, Xue W, Yang S: **Chemical constituents of *Caesalpinia decapetala* (Roth) Alston.** Molecules 2013; **18**:1325-1336.

6. Atta-ur-Rahman, Zaman K, Perveen S, Habib-ur-Rehman, Muzaffar A, Choudhary M, Pervin A. **Steroidal alkaloids from leaves of *Buxus sempervirens***. Phytochemistry 1991; **30**:1298-1293.
